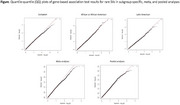# Gene‐based association study of rare structural variants in Alzheimer's Disease

**DOI:** 10.1002/alz70855_104615

**Published:** 2025-12-24

**Authors:** Songmi Lee, Adam C English, Rui Xia, Gina M. Peloso, Josh Bis, Honghuang Lin, Seung Hoan Choi, Nancy Heard‐Costa, Anita L. DeStefano, Fritz J Sedlazeck, Myriam Fornage

**Affiliations:** ^1^ University of Texas Health Science Center at Houston, Houston, TX, USA; ^2^ Baylor College of Medicine Human Genome Sequencing Center, Houston, TX, USA; ^3^ Boston University, Boston, MA, USA; ^4^ University of Washington, Seattle, WA, USA; ^5^ University of Massachusetts Chan Medical School, Worcester, MA, USA; ^6^ Department of Biostatistics, Boston University School of Public Health, Boston, MA, USA; ^7^ Boston University Chobanian & Avedisian School of Medicine, Boston, MA, USA; ^8^ Boston University School of Public Health, Boston, MA, USA

## Abstract

**Background:**

Structural variants (SV) are genomic alterations > 50 base pairs that can impact gene expression and protein function. However, their role in Alzheimer's Disease (AD) remains unclear. Using a novel SV calling pipeline, we identified SVs with high accuracy in a diverse sample from the Alzheimer's Disease Sequencing Project (ADSP) and investigated the gene‐based association of rare SVs with AD.

**Methods:**

We included samples from ADSP 17K whole genome sequence data. SV calling was performed using Biograph, which leverages assembly methods and graph‐based representation. After quality filtering, we identified 194,744 deletions, 151,858 insertions, and 10,388 inversions with minor allele frequency (MAF) < 1% in 11,890 individuals (5,585 AD cases; 6,305 controls). Ancestry was inferred using Grafpop. We performed gene‐based association analyses of rare SVs with AD using a logistic mixed‐effects model in a hybrid of burden test and SKAT implemented in GMMAT, incorporating PhenoSV annotation scores and MAF as weights. Subgroup‐specific and meta‐analyses were conducted, along with pooled analyses across the total samples. We adjusted for sex, technical covariates, SV principal components, and relatedness. Statistical significance was evaluated using a Bonferroni‐corrected threshold based on the number of genes (*p* < 6 x 10^‐6^).

**Results:**

Based on genetic similarity, the study participants were classified into four population subgroups including 6,327 (53%) European (EUR), 3,371 (28%) African or African American (AFR), 2,126 (18%) Latin American (LAT), and 66 (<1%) others. No gene‐based tests achieved Bonferroni‐corrected statistical significance; however, a few suggestive associations were observed (*p* < 5 x 10^‐4^) (Figure). The EUR subgroup analyses showed an association with AD in *ACLY* (*p* = 2 x 10^‐4^), which encodes a protein involved in the biosynthesis of acetylcholine. *PARP10* (*p* = 1.7 x 10^‐4^) and *ENDOV* (8.4 x 10^‐5^) showed suggestive associations with AD in the AFR and LAT subgroup analyses, respectively. The meta‐analyses showed that the top gene associated with AD was *RPL8* (*p* = 9 x 10^‐4^). The pooled analyses revealed an association in *SSPN* (*p* = 3 x 10^‐4^) with AD, which encodes a protein found in the neuromuscular junction.

**Conclusion:**

Our findings suggest a role of rare SVs in AD. Expanded sample sizes are needed for confirmation.